# Clinicopathologic Features and Treatment of CD10-Positive Mantle Cell Lymphoma: A Case Report and Review of Literature

**DOI:** 10.3389/pore.2022.1610588

**Published:** 2022-08-25

**Authors:** Christopher Hino, Bryan Pham, Austin L. Gray, Jun Wang, Dan Ran Castillo, Mojtaba Akhtari, Yan Liu

**Affiliations:** ^1^ Department of Internal Medicine, Loma Linda University Medical Center, Loma Linda, CA, United States; ^2^ Department of Pathology, Loma Linda University, Loma Linda, CA, United States; ^3^ Department of Oncology/Hematology, Loma Linda University, Loma Linda, CA, United States

**Keywords:** case report, mantle cell lymphoma, CD10, CCND1, B-cell chronic lymphoproliferative disorders, immunophenotyping

## Abstract

Mantle cell lymphoma (MCL) is a rare and aggressive non-Hodgkin’s B cell lymphoma characterized by the translocation t(11;14) (q13;32) and overexpression of *CCND1*. MCL is immunophenotypically identified as CD20^+^, CD5^+^, CyclinD1+, CD43^+^, CD10^−^, BCL6^−^, and CD23^−^. It is often distinguished from B cell lymphomas of germinal center cell origin by the absence of CD10 expression. Here we report the unique clinicopathologic features of a patient with CD10^+^ MCL with gastrointestinal involvement and review current literature identifying this unique immunophenotype.

## Introduction

Mantle cell lymphoma (MCL) is a rare and aggressive form of non-Hodgkin’s lymphoma (NHL) characterized by the abnormal proliferation of mature B lymphocytes. The disease, which accounts for 3–10% of all adult-onset NHL, presents predominately in men with a median age of 68 years (1, 2). Most cases are diagnosed at an advanced Ann Arbor stage and often manifest as progressive generalized lymphadenopathy, cytopenia, splenomegaly, and symptomatic extra-nodal involvement (1, 3).

MCL is genetically characterized by the translocation t(11;14) (q13;q32), which results in the overexpression of the proto-oncogene *CCND1* caused by fusion to an enhancer of the immunoglobulin heavy chain (IgH) gene (1). This translocation event leads to the constitutive overexpression of cyclin D1 and is believed to be the primary pathogenic factor driving the dysregulated proliferation of pre-germinal-center B cells in the mantle zone areas of lymphoid follicles (4, 5). Morphologically, classical or nodal MCL (cMCL) is characterized by small to medium sized lymphocytes with irregular nuclei and inconspicuous nucleoli and small amount of cytoplasm arising from naïve-like mature B cells which express the oncogenic transcription factor SOX11. Comparatively, the more rare leukemic non-nodal MCL (nnMCL) is derived from SOX11-negative memory-like B cells (6, 7).

Most MCL cases are believed to originate from a CD20^+^ CD5^+^ CD43^+^ naïve-pre-germinal B cell population. MCL does not usually express the germinal center (GC) associated antigens such as CD10 and BCL-6 that are thus used to distinguish MCL from B cell lymphomas of germinal center origin, including follicular lymphoma, Burkitt lymphoma, and a subset of diffuse large B cell lymphoma, in addition to a subset of lymphoblastic lymphoma/leukemias (8). To date, few CD10^+^ MCLs have been reported in the literature (9–11). Here we describe the clinical features of a patient uniquely presenting with CD10^+^ cMCL and review current literature which have identified this distinctive immunophenotype.

## Case Report

A 73- year-old male who underwent evaluation for normocytic anemia and microscopic hematuria was incidentally found to have multiple enlarged mesenteric lymph nodes throughout the abdomen and upper pelvis, with the largest measuring 1.2 cm in short axis. At the initial presentation, the patient denied fevers, chills, night sweats, weight loss, or abdominal pain. His clinical examination revealed no notable hepatosplenomegaly, or palpable lymphadenopathy. His lab results were significant for a hemoglobin of 10 g/dl (down from 13 one year ago), but were otherwise unremarkable. A peripheral smear at the time demonstrated no circulating blasts, or overt dysplastic features. However, a repeat CT abdomen and pelvis 10 months later demonstrated increased size and number (>50) of multiple enlarged mesenteric lymph nodes, with the largest having a confluent dimension of 3 cm.

**FIGURE 1:**
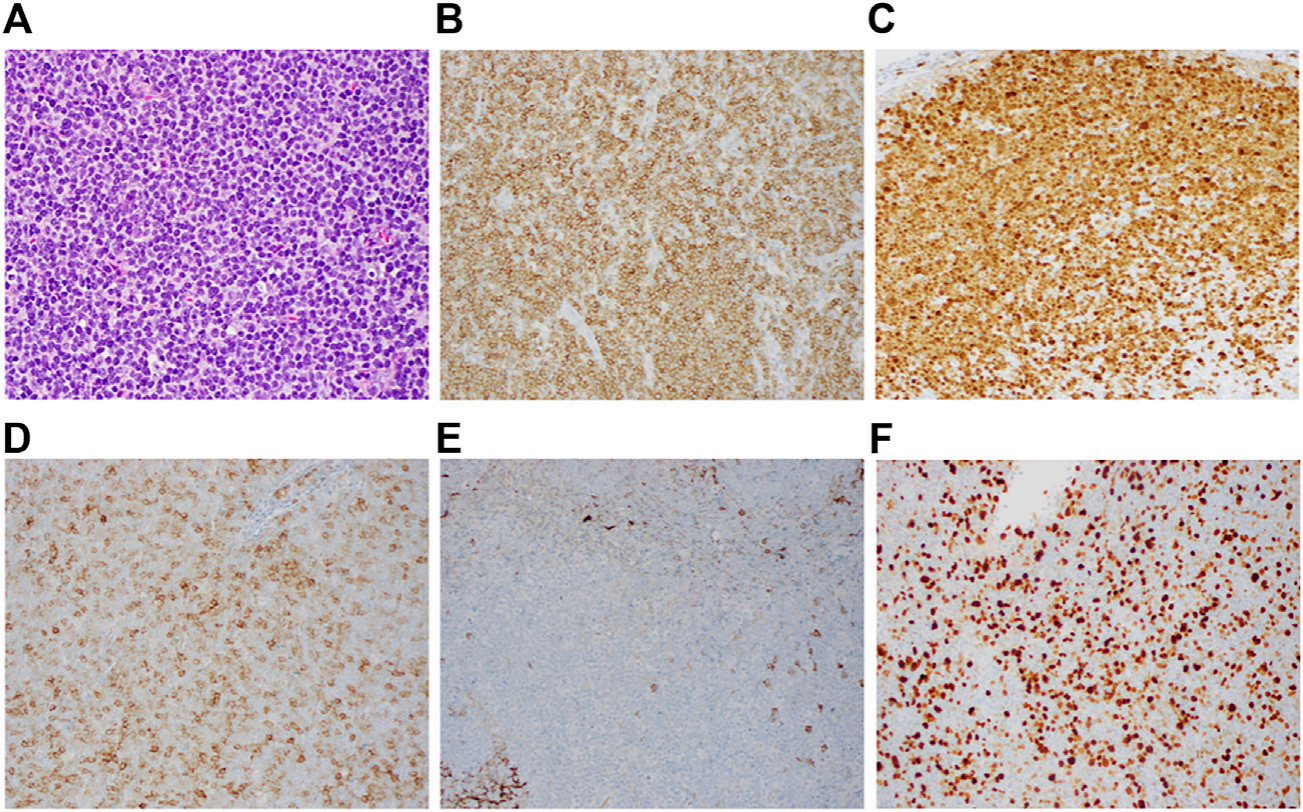
Immunohistochemistry of CD10^+^ mantle cell lymphoma. Representative lymph node biopsy demonstrates **(A)** morphologic features of the mantle cell lymphoma (H&E, x1,000) with immunostaining **(B)** positive for CD5, **(C)** positive for cyclin D1, **(D)** Variabily positive for CD10, **(E)** negative for CD23, **(F)** Elevated Ki-67 proliferation index (Overall 40%–70%).

**FIGURE 2:**
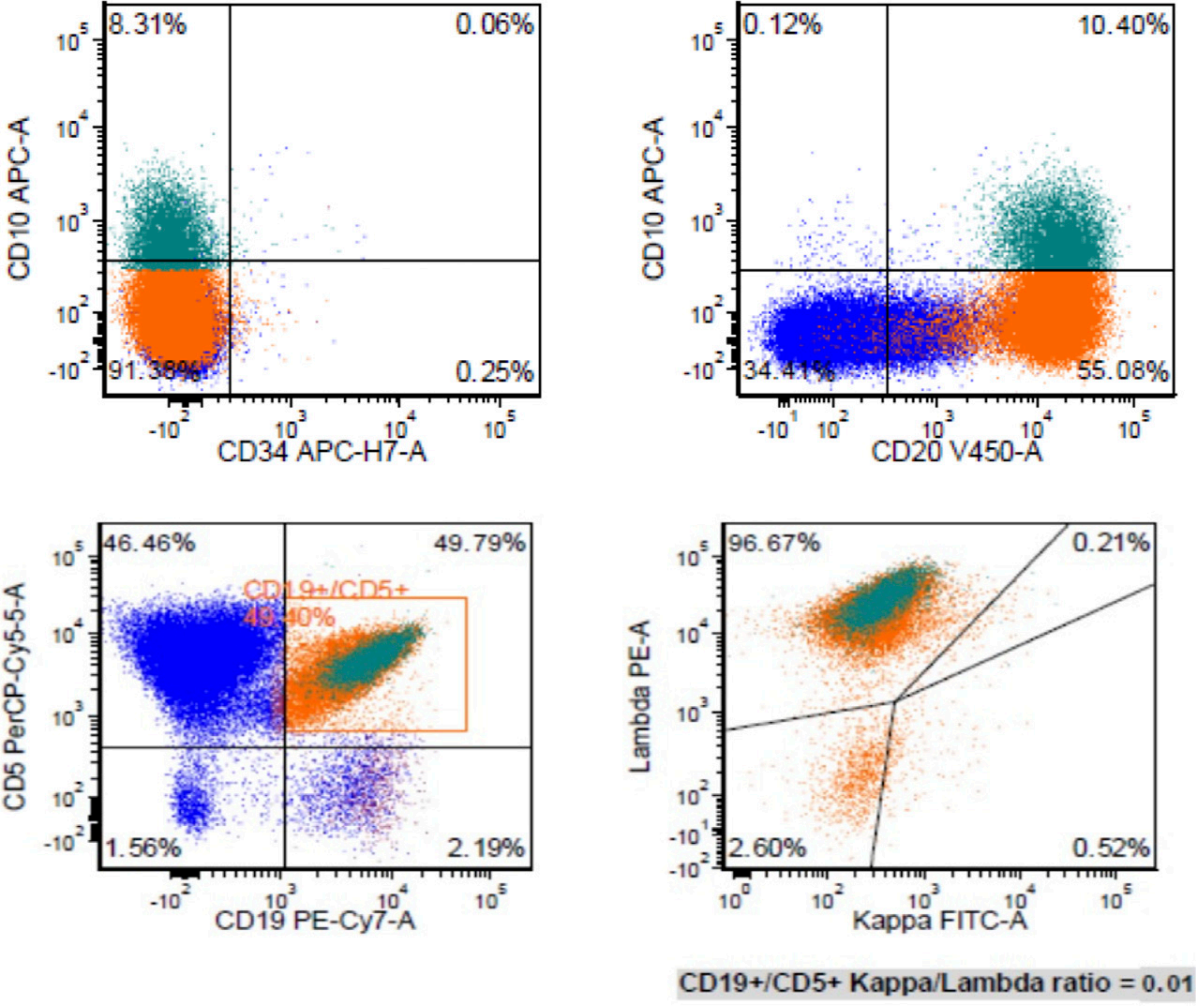
Flow cytometric analysis of bone marrow aspirate revealed a monotypic lambda restricted CD5+/CD10+ B cell population.

A mesenteric lymph node biopsy was obtained, which revealed total effacement by small to medium-sized lymphocytes demonstrating an irregular nuclear border, clumped chromatin, and inconspicuous nucleoli and small amount of cytoplasm. Immunohistochemical (IHC) staining of the biopsied node demonstrated positive expression for CD5, CD20, CD79a, PAX5, BCL-2, SOX11, Cyclin-D1 and negative for CD3, CD15, CD23, CD30, and BCL-6; therefore, confirming the diagnosis of MCL, with a mean Ki-67 index 55% (40–70%). Remarkably, CD10 was found to be variably positive in about 70% of tumor cells (Figure 1). Bone marrow biopsy demonstrated scattered atypical B cells positive for CD5 and CD20 with lambda restriction, compatible with bone marrow involvement by mantle cell lymphoma. Flow cytometric analysis of the bone marrow aspirate confirmed the presence of a monotypic, lambda restricted, CD5+/CD10 dim + B cell population (Figure 2).

**FIGURE 3:**
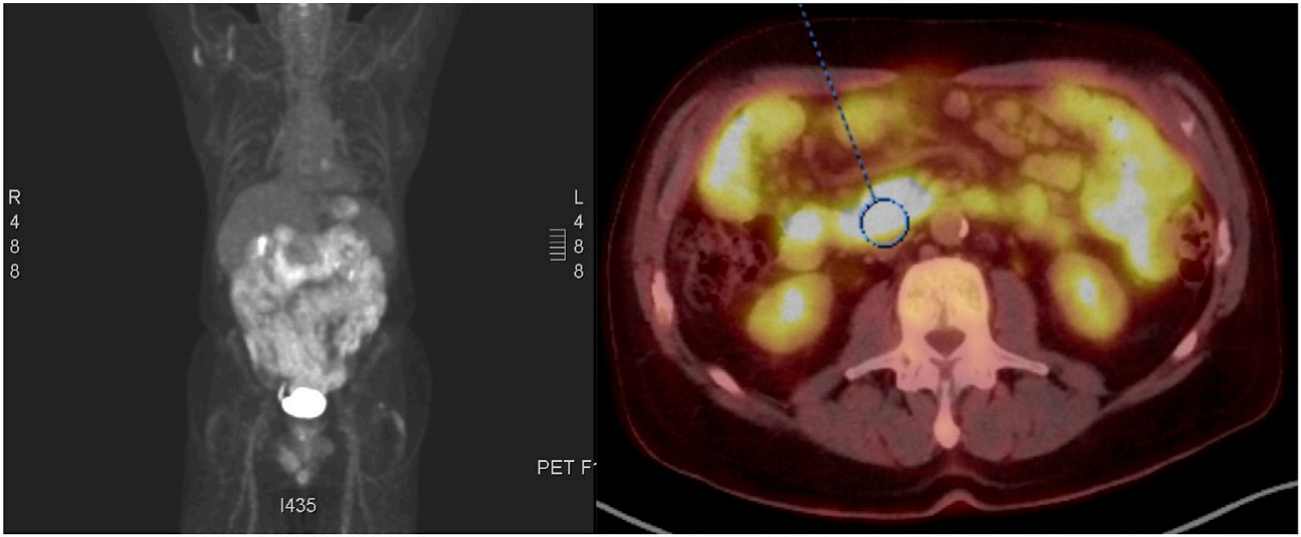
PET-CT demonstrating hypermetabolic , diffuse wall thickening of the stomach and throughout the small bowel.

A positron emission tomography (PET) scan showed hypermetabolic, diffuse wall thickening of the stomach and numerous enlarged mesenteric lymph nodes/diffuse hypermetabolic activity throughout the small bowel (Figure 3). Based on the PET/CT findings, the patient underwent an esophagogastroduodenoscopy and colonoscopy. Diffuse friable, edematous, and erythematous mucosa was observed within the gastric body, greater curvature of the gastric antrum, and second part of the duodenum. Two sessile polyps were biopsied in the proximal sigmoid colon at the cecum. Immunohistochemical stains of the duodenal and proximal sigmoid colonic biopsies showed large aggregates of small to medium-sized B lymphocytes, often with irregular nuclei, expressing cyclin D1 with 40%–70% expression of the proliferative antigen, Ki-67. These findings were consistent with a diagnosis of MCL with gastrointestinal involvement. FISH analysis further confirmed the presence of t(11;14) without 17p deletion, and the absence of t(14;18) translocation. The disease was classified as stage IV with GI involvement according to the Ann Arbor staging system, MCL international prognostic index (MIPI) score was 7.6.

The patient was initiated on induction treatment with daily administration 560 mg Ibrutinib (dose reduced to 420 mg due to hypertension) + 20 mg Venetoclax combined with escalating doses of Venetoclax to 400 mg over the first 5 weeks (12). Upon completion of 18 weeks of treatment, a repeat PET/CT showed complete remission while a bone marrow biopsy and aspiration demonstrated 30% cellularity with no evidence of lymphoma. He subsequently received autologous peripheral blood stem cell transplant (PBSCT) with successful engraftment. At follow-up a repeat colonscopy, endoscopy, and PET/CT confirmed that patient had achieved complete remssion.

## Discussion

For many years, MCL was largely considered an incurable disease, with a median overall survival of 3–5 years (13). Standard therapy for patients with MCL consists of chemotherapy: R-CHOP regimen, consisting of cyclophosphamide, doxorubicin, vincristine, and prednisone combined with rituximab. However, the recent introduction of novel chemo immunotherapies in the last decade has dramatically transformed the prognostic and therapeutic landscape of MCL (14). In particular, the development of BTK inhibitors (ibrutinib, acalabrutinib, and zanubritinib), Bcl-2 antagonists (Venetoclax), and proteasome inhibitors (bortezomib) have revolutionized MCL management (15).

Recent genomic and epigenomic studies have considerably improved our understanding of the pathogenesis of MCL and demonstrated the remarkable complexity of the MCL microenvironment (7). While the t(11;14) (q13;q32) translocation leading to the overexpression of CCND1 is believed to be the primary event in the pathogenesis of the tumor, the acquisition of additional mutations in cell cycle dysregulation, DNA damage response, or cell survival pathways are important contributors to the high degree of genomic instability observed in MCL (16, 17). This in turn reflects the unique heterogeneity of clinical presentation and response to therapy. It further highlights the need to fully elucidate the clinicopathologic features that define subpopulations of MCL.

MCL classically expresses the mature B cell markers CD19, CD20, CD79a and are often distinguished from other B cell lymphomas by the expression of cyclin D1, CD5, FMC7, CD43 and the absence of CD10 and CD23 (11, 18). The case presented here describes a unique CD10 expressing MCL subpopulation that has rarely been identified in the literature. Our current understanding of CD10^+^ MCL is based on a limited number of case reports and studies, totaling less than 80 known cases in the literature (9–11, 19–23).

Despite the usual uniform morphology and immunophenotype, it is well recognized that cases of MCL may display clinical and molecular heterogeneity (24–29). So-called aberrant phenotypes have been described, such as CD10-positive MCL. CD10 is a transmembrane glycoprotein, normally expressed in early lymphoid progenitors and normal GC cells and widely used in lymphoma diagnosis. However, the aberrant expression of CD10 by immunohistochemistry and flow cytometry has been reported in a few cases of well-studied MCL and is believed to contribute to an even worse prognosis.

The pathologic significance of aberrant CD10 expression in MCL is highlighted by the fact that CD10 is normally expressed in early lymphoid progenitors and germinal center B cells. This raises the possibility that some MCL subpopulations may either 1) be derived from germinal center B cells, 2) be influenced by the germinal center microenvironment or 3) acquire CD10 expression through somatic mutation (24, 30). Co-expression of a second germinal center associated antigen, BCL6, has also been reported in a fraction of CD10^+^ MCL cases; supporting the hypothesis that at least a subset of MCL may be derived from germinal center B cells (10, 11). However, others have observed that classical MCL that transformed to blastoid MCL acquired CD10 expression at the time of transformation. In the present case, our patient was found to be CD10^+^ and BCL6-at the time of diagnosis with classical MCL. Previous studies have observed aberrant co-expression of Bcl-6 and CD10 in MCL (9, 10, 20). Considering that Bcl-6 expression is predominately restricted to germinal center B cells, the absence of BCL-6 in the present case may indicate that these cells did not arise from germinal center B cells (31).

Due to the paucity of reported CD10^+^ MCL cases, the correlation between CD10^+^ expression and clinical outcome has been studied to a very limited extent. A recent study by Xu et al. 2018 compared the clinicopathologic features between 30 patients with CD10^+^ MCL versus CD10-negative MCL patients. They had found that while CD10 expression was not independently associated with a difference in overall survival, CD10 expression was associated with an even worse prognosis in patients with a high Ki-67 (>60%), blastoid/pleomorphic morphology, or high MIPI (9). Remarkably, the patient described above with high MIPI score (7.6) and Ki-67 40%–70% was able to achieve complete response with allogeneic HSCT following Ibrutinib and ventoclax chemotherapy. The combination of the BTK inhibitor Ibrutinib and BCL2 inhibitor ventoclax has been demonstrated to have superior efficacy in patient’s primarily with relapsed/refractory MCL than those who received either Ibrutinib or ventoclax monotherapy (12). However, in the present case we show that use of combination therapy of ibrutinib and ventoclax may also be an effective therapeutic strategy in untreated cases with CD10^+^ phenotype. Given promising therapeutic response in the present case, future studies should further investigate how CD10^+^ MCL responds to this novel combination therapy.

## Conclusion

Although CD10^+^ MCL is rare, it should be included in the differential diagnosis of CD10 + B cell lymphomas. Growing evidence suggests that CD10^+^ MCL is a molecularly distinct entity that requires further evaluation. Uncovering the origin of this unique subpopulation may help us to better understand the pathogenesis of MCL and spark the development of novel immunotherapies for this rare and aggressive lymphoma.

## Patient Perspective

At 2 months follow-up, the patient and his family expressed satisfaction with treatment and subsequent remission.

## Data Availability

The original contributions presented in the study are included in the article/supplementary material, further inquiries can be directed to the corresponding author.
